# Chondroitin and glucosamine sulphate reduced proinflammatory molecules in the DRG and improved axonal function of injured sciatic nerve of rats

**DOI:** 10.1038/s41598-022-06554-4

**Published:** 2022-02-24

**Authors:** Olutayo Folajimi Olaseinde, Bamidele Victor Owoyele

**Affiliations:** Neuroscience and Inflammation Unit, Department of Physiology, College of Health Sciences, University of Ilorin, Ilorin, Kwara Nigeria

**Keywords:** Biochemistry, Molecular biology, Neuroscience, Physiology, Anatomy, Diseases

## Abstract

Neuropathic pain (NP) is an abnormality resulting from lesion or damage to parts of the somatosensory nervous system. It is linked to defective quality of life and often poorly managed. Due to the limited number of approved drugs, limited efficacy and side effects associated with the approved drugs, drugs or drug combinations with great efficacy and very minimal or no side effects will be of great advantage in managing NP. This study aimed at investigating the synergistic antinociceptive effects of the combination of glucosamine sulphate (GS) (240 mg/kg) and chondroitin sulphate (CS) (900 mg/kg) in chronic constriction injury (CCI)-induced neuropathy in rats. Forty-two Wistar rats were randomly distributed into seven groups (n = 6). Sciatic nerve was ligated with four loose ligatures to induce NP. Effects of drugs were examined on stimulus and non-stimulus evoked potentials, expression of dorsal root ganglia (DRG) pain modulators and structural architecture of DRG. Oral administration of GS and CS for 21 days reduced hyperalgesia, allodynia, sciatic nerve functional aberration and DRG pain modulators. Histopathology and immunohistochemistry revealed restoration of structural integrity of DRG. Our result showed that the combination of GS and CS produced antinociceptive effects by attenuating hyperalgesia, allodynia and downregulation of NP mediators. GS and CS additionally produced synergistic analgesic effect over its individual components.

## Introduction

Neuropathic pain (NP) is described as pain caused by an injury or abnormality of the somatosensory nervous system^1^. About 7–10% of the whole global population is affected by this debilitating condition^2^. About a fourth of people with diabetes and a third of people with HIV have neuropathic pain^3^. A recent systematic review of medicines for NP by international association for the study of pain (IASP) highlighted drugs that are in use for the treatment of ailment and these comprised of tricyclic antidepressants (TCAs), cannabinoids and serotonin and noradrenaline reuptake inhibitors (SNRIs). Most of the studies conducted in the systemic review were done in diabetic neuropathy or postherpetic neuralgia with no main focus on CCI of sciatic nerve. It was also reported that data did not identify one particular drug class that is superior in any particular neuropathic pain. Due to the limited number of approved drug and the efficacy of these drugs, about half of patients with NP takes concomitantly two or more drugs in managing this condition^4^, thereby resulting to compliance issues. Reports have shown that drug combinations have given better results in the management of chronic neuropathic pain^5^. The frequently prescribed drugs in the management of NP such as TCAs, SNRIs and cannabinoids show moderate efficacy of at most 50% pain relief in about a third of the patients^6^. Also, many dose limiting side effects have been associated with these drugs.

Pain mechanism is a network of knotty and constantly changing systems of sensory, cognitive and behaviour that evolved into a synchrony of protective responses to arriving noxious stimuli or stimuli that portends tissue damage or survival^7^. Glucosamine sulphate (GS) and chondroitin sulphate (CS) are compounds identified for osteoarthritis treatment. These compounds are also referred to as glycosaminoglycans (GAGs) and naturally present in the body where they form essential components of proteoglycans^8–10^. GAGs have been reported to possess anti-inflammatory, antioxidant and chondroprotective properties^10^. Hence, the study has therefore been conducted to investigate the synergistic effect of the combination of chondroitin sulphate and glucosamine sulphate treatment on dorsal root ganglia inflammatory markers in CCI neuropathic pain in male Wistar rats.

## Results

### Pre-surgical (baseline) behavioural and validatory post-surgical behavioural tests

None of the animals showed any symptoms of cold allodynia, thermal hyperalgesia or mechanical allodynia in the ipsilateral paws prior to the surgical procedure. The sciatic nerve functional index measurements were normal across all the groups prior to surgical intervention. There were no significant differences between the baseline measurements in all the various tests prior to surgical intervention. Following surgical (CCI) intervention, the animals exhibited behavioural changes different from the baseline measurements, normal and sham control groups immediately the animals recovered from sedation effect of the anaesthetic drug. These behavioural changes were characterised by protective behaviour of the ipsilateral hind paw due to developed spontaneous pain stimulus. All the rats with CCI intervention exhibited mechanical allodynia, thermal hyperalgesia, cold allodynia and sciatic nerve functional derangement (measured via the sciatic nerve functional index). These behavioural changes were observed to be statistically (*p* < 0.05) different from the normal and sham control groups. These observations were made on the third day following CCI intervention.

### GS and CS reversed mechanical allodynia

**Figure 1:**
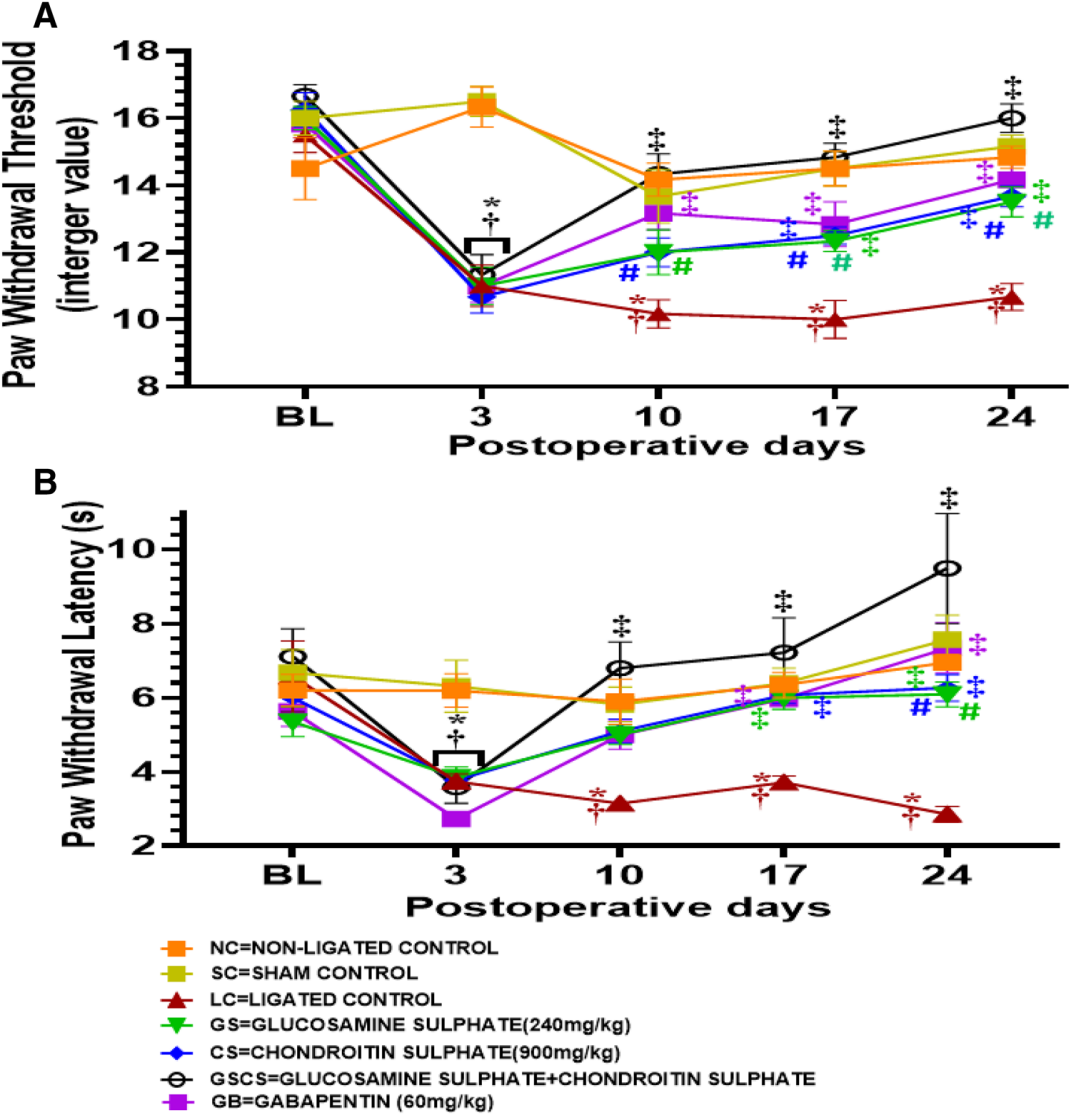
GS and CS reversed both mechanical allodynia and thermal hyperalgesia in sciatic nerve CCI model of neuropathic pain. (**A**) Mechanical allodynia test (**B**) Thermal analgesia tests. Values are shown as the mean ± SEM (n = 6 for each group) (*†‡# *p* < 0.05. *Significant versus NC, **†**Significant versus SC, **‡** Significant versus LC. **#**Significant versus GSCS. BL:baseline threshold).

observations from this study showed that there was mechanical allodynia on the third day after CCI intervention. The mechanical allodynia was characterised by paw withdrawal from the appropriate von Frey filaments. The mechanical allodynia observed was statistically significant (*p* < 0.05) till the 24th day in the ligated control (LC) group (11.00 ± 0.62) compared to the normal (16.33 ± 0.61) and sham control groups (16.50 ± 0.45) (Fig. 1A). However, GS significantly (*p* < 0.05) reduced the observed mechanical allodynia on the 17th day post-surgery (12.33 ± 0.31 vs 10.00 ± 0.56) and the 24th day post-surgery (13.50 ± 0.45 vs 10.67 ± 0.40) compared to ligated control. CS significantly (*p* < 0.05) reduced the observed mechanical allodynia on the 17th day post-surgery (12.50 ± 0.26 vs 10.00 ± 0.56) and the 24th day post-surgery (13.67 ± 0.31 vs 10.67 ± 0.40). This was characterised by a significant increase in the paw withdrawal threshold (PWT) of the GS and CS treated rats as (Fig. 1A). Interestingly, the combination of GS and CS showed a more significant reduction (p < 0.05) in mechanical allodynia compared to the LC animals from the 10th (14.33 ± 0.60 vs 10.17 ± 0.42), 17th (14.83 ± 0.42 vs 10.00 ± 0.56), 24th (16.00 ± 0.43 vs 10.67 ± 0.40) day post-CCI. This is characterised by an increase in PWT of the animals treated with the combination of GS and CS. Furthermore, the combination of GS and CS showed a synergistic and significant (*p* < 0.05) reduction in mechanical allodynia when compared when the two agents were administered singly. Animals treated with Gabapentin (GB) (reference drug) also showed a significant (*p* < 0.05) reduction in mechanical allodynia compared to the LC animals on the 10th (13.17 ± 0.49 vs 10.17 ± 0.42), 17th (12.83 ± 0.67 vs 10.00 ± 0.56) and 24th (14.17 ± 0.49 vs 10.67 ± 0.40) day post-CCI (Fig. 1A).

### GS and CS reversed thermal hyperalgesia

The results showed that thermal hyperalgesia, characterised by paw withdrawal latency from hot plate apparatus, was recognised on the third day after CCI intervention. Thermal hyperalgesia was significantly sustained till the 24th day following CCI (Fig. 1B). It was observed that thermal hyperalgesia was progressive in intensity with days. On the 3rd day post-CCI, thermal hyperalgesia in LC group was significantly (*p* < 0.05) different from those of the normal and sham control groups, from the 3rd to the 24th day. However, animals treated differently with GS showed a significant (*p* < 0.05) reduction in thermal hyperalgesia on the 17th (5.99 ± 0.31 vs 3.70 ± 0.17) and 24th (6.09 ± 0.33 vs 2.85 ± 0.21) day post CCI compared to the LC group. Also, animals treated differently with CS showed a significant (*p* < 0.05) reduction in thermal hyperalgesia on the 17th (6.07 ± 0.22 vs 3.70 ± 0.17) (*p* < 0.05) and 24th (6.26 ± 0.36 vs 2.85 ± 0.21) day post CCI. This was characterized by a withdrawal latency increase as shown in Fig. 1B. The combination of GS and CS showed a significant reduction in thermal hyperalgesia from the 10th (6.80 ± 0.71 vs3.14 ± 0.13), 17th (7.22 ± 0.14 vs 3.17 ± 0.17) and 24th (9.49 ± 1.48 vs 2.85 ± 0.21) day post-CCI compared to the LC group. Interestingly, the animals treated with the combination of GS and CS showed a significant reduction in thermal hyperalgesia on the 24th day post-CCI (9.49 ± 1.48 vs 2.85 ± 0.21) compared to animals treated with GS (6.09 ± 0.33 vs 2.85 ± 0.21) and CS (6.26 ± 0.36 vs 2.85 ± 0.21) individually. Animals treated with GB also showed a significant (*p* < 0.05) reduction in thermal hyperalgesia compared to the LC animals on the 17th (5.97 ± 0.06 vs 3.17 ± 0.17) and 24th (7.34 ± 0.68 vs 2.85 ± 0.21) day post-CCI (Fig. 1B).

### GS and CS reversed cold allodynia

**Figure 2:**
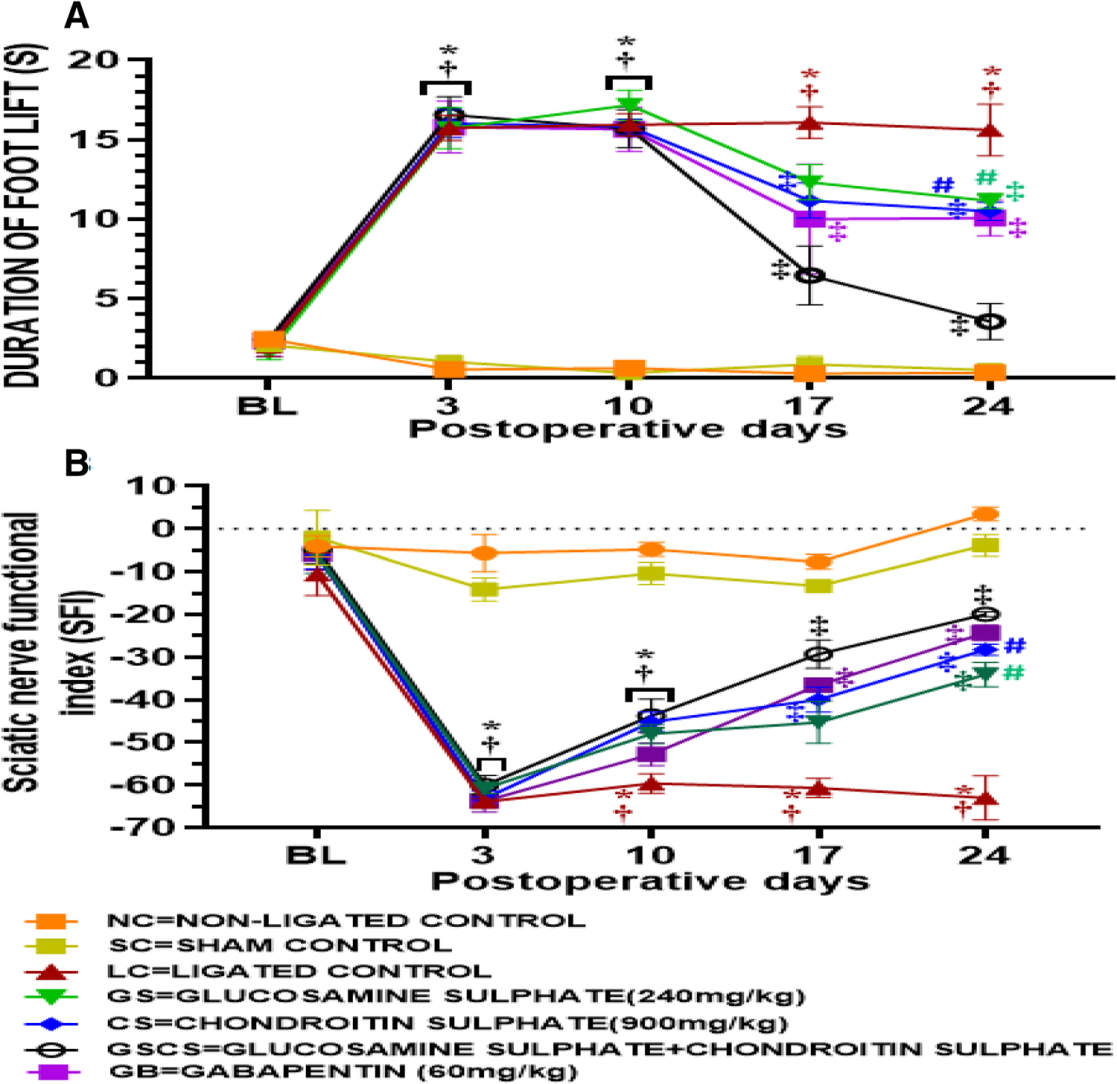
GS and CS reversed cold allodynia and SFI in sciatic nerve CCI model of neuropathic pain. (**A**) Cold allodynia test (**B**) SFI. Values are shown as the mean ± SEM (n = 6 for each group) (*†‡# p < 0.05. *Significant versus NC, **†**Significant versus SC, **‡**Significant versus LC., **#**Significant versus GSCS. BL:baseline threshold).

this study showed that cold allodynia characterised by increase in the duration of foot lift (DFL) to acetone drop test was observed in the LC animals and this was recognised from the 3rd day of CCI intervention and significantly sustained till the 24th day of CCI intervention (Fig. 2A). Animals treated with GS significantly (*p* < 0.05) reduced DFL to acetone test on the 17th (12.31 ± 1.12 vs 16.07 ± 0.99) and 24th (11.15 ± 0.47 vs 15.61 ± 1.63) day post-CCI compared to the LC animals. Animals treated with CS significantly (*p* < 0.05) reduced DFL to acetone test on the 17th (11.15 ± 1.10 vs 16.07 ± 0.99) and 24th (10.49 ± 0.57 vs 15.61 ± 1.63) day post-CCI compared to the LC animals. The combination of GS and CS treatment brought about a significant (*p* < 0.05) reduction in DFL compared to the LC group on the 17th (6.46 ± 1.85 vs 16.07 ± 0.99) and 24th (3.56 ± 1.13 vs 15.61 ± 1.63) day post CCI. The combination of GS and CS treatment also brought about a synergistic and significant (*p* < 0.05) reduction in DFL on the 17th (6.46 ± 1.85 vs 12.31 ± 1.12 and 11.15 ± 1.10) and 24th (6.46 ± 1.85 vs 11.15 ± 0.47 and 10.49 ± 0.57) day post-CCI when compared to when GS and CS were administered singly (Fig. 2A). Animals treated with GB also showed a significant (*p* < 0.05) reduction in DFL compared to the LC animals on the 17th and 24th day post-CCI (Fig. 2A).

### GS and CS improved Sciatic nerve functional index (SFI)

It was observed from this study that the baseline measurement of SFI showed no significant difference. However, following CCI intervention, there was a significant (*p* < 0.05) decrease in SFI (− 63.97 ± 0.73) of ligated animals compared to normal (− 5.67 ± 4.36) and sham (− 14.17 ± 2.73) control groups (Fig. 2B). Animals treated with GS significantly (*p* < 0.05) increased SFI on the 24th (− 34.12 ± 2.84 vs − 63.04 ± 5.17) day post-CCI compared to the LC animals. Also, Animals treated with CS significantly (p < 0.05) increased SFI on the 24th (− 28.29 ± 1.29 vs − 63.04 ± 5.17) day post-CCI compared to the LC animals. The combination of GS and CS treatment brought about a significant (*p* < 0.05) increase in SFI compared to LC group on the 17th (− 29.34 ± 3.32 vs − 60.75 ± 2.23) and 24th (− 19.96 ± 0.59 vs − 63.04 ± 5.17) day post-CCI. GS and CS combination brought about a synergistic significant (*p* < 0.05) increase in SFI (− 19.96 ± 0.59) on the 24th day post-CCI when compared to administration of GS (− 34.12 ± 2.84) and CS (− 28.29 ± 1.29) individually (Fig. 2B).

### GS and CS reversed NP modulators in the DRG

**Figure 3:**
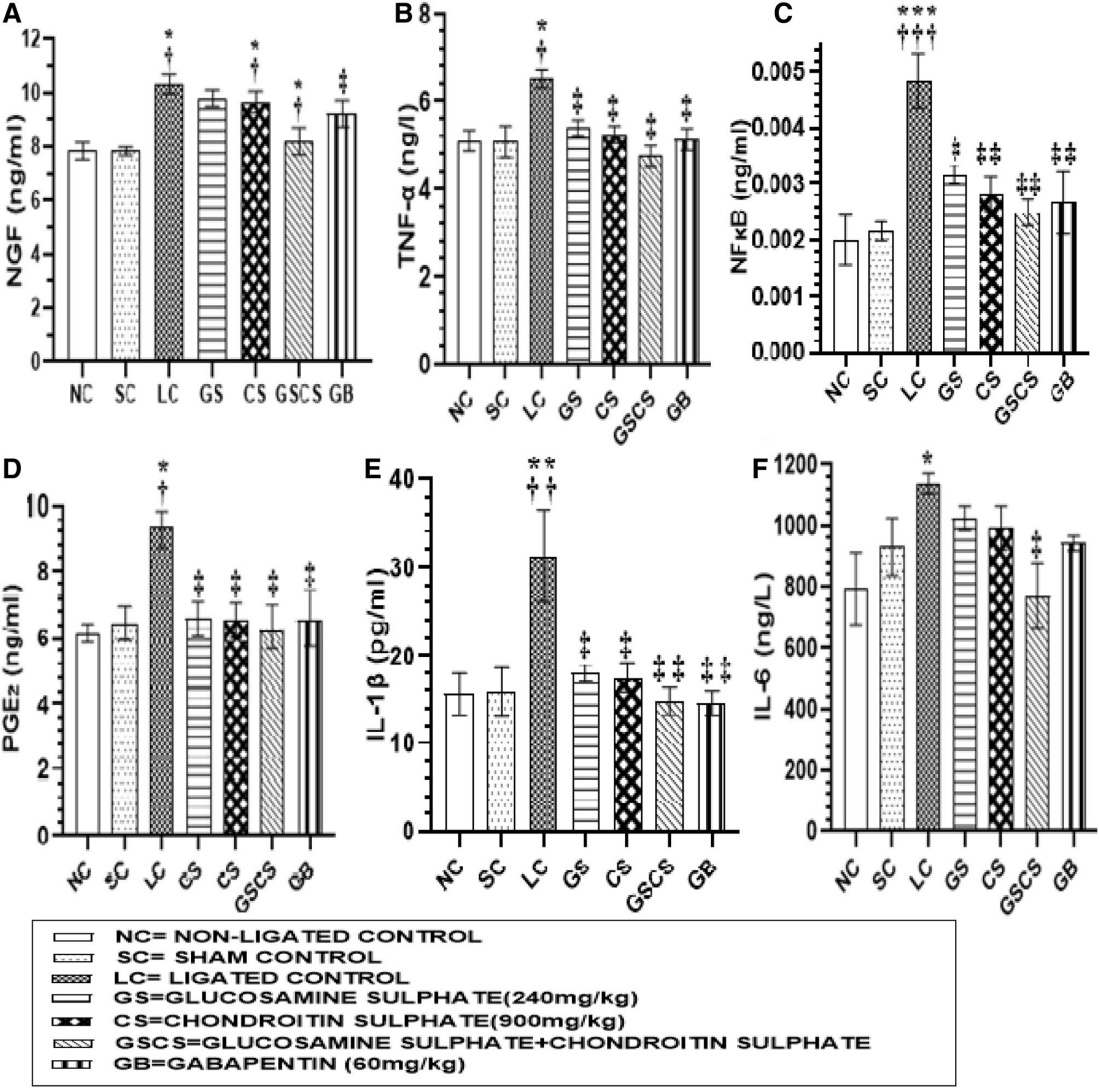
GS and CS reversed proinflammatory mediators in sciatic nerve CCI model of neuropathic pain. (**A**) NGF (**B**) TNF-α (**C**) NFκB (**D**) PGE_2_ (**E**) IL-1β (**F**) IL-6. Values are shown as the mean ± SEM (n = 6 for each group) (*†‡*p* < 0.05, **††‡‡*p* < 0.001, ***†††‡‡‡ *p* ≤ 0.0009 ≥ 0.0001. *Significant versus NC., **†**Significant versus SC., **‡**Significant versus LC).

The concentration of NGF measured in the LC group was significantly (*p* < 0.05) increased (10.32 ± 0.37) compared to normal (7.83 ± 0.32) and sham (7.84 ± 0.32) control groups, while the concentration of NGF in the DRG of GS (8.80 ± 0.23) and CS treated groups was significantly (*p* < 0.05) reduced (8.82 ± 0.22 vs 10.32 ± 0.37) compared to LC group (Fig. 3A). The combination of GS and CS significantly (p < 0.05) reduced NGF concentration (8.18 ± 0.50 vs 10.32 ± 0.37) in the DRG compared to the LC group (Fig. 3A). GB treated animals also showed a significant (*p* < 0.05) reduction in the concentration of NGF in the DRG (8.65 ± 0.20 vs 10.32 ± 0.37) compared to LC group. The concentration of TNF-α measured in the DRG of LC animals was significantly (*p* < 0.0.05) increased (6.53 ± 0.20) compared to normal (5.12 ± 0.24) and sham (5.09 ± 0.36) animals (Fig. 3B.). Animals treated with GS (5.41 ± 0.18) and CS (5.26 ± 0.20) showed a significant reduction in the level of TNF-α concentration in the DRG compared to LC (6.53 ± 0.20) animals. The combination of GS and CS treatment produced a significant (*p* < 0.05) reduction in the TNF-α level of DRG (4.76 ± 0.23 vs 6.53 ± 0.20) when compared to LC group (Fig. 3B). GB also brought about a significant (*p* < 0.0.05) reduction in TNF-α level in the DRG (5.15 ± 0.25 vs 6.53 ± 0.20) compared to LC group (Fig. 3B). Evaluation of the DRG level of NFκB showed a significant (*p* < 0.05) increase in the LC group (0.0048 ± 0.0005) compared to sham (0.002 ± 0.0004) and non-ligated (0.002 ± 0.0004) control groups (Fig. 3C). However, GS treatment significantly (*p* < 0.05) reduced DRG level of NFκB (0.0032 ± 0.00017 vs 0.0048 ± 0.0005) compared to the LC group. CS treatment significantly (*p* < 0.05) reduced DRG level of NFκB (0.0028 ± 0.00075 vs 0.0048 ± 0.0005) compared to the LC group. The combination of GS & CS treatment significantly (*p* < 0.05) reduced DRG level of NFκB (0.0025 ± 0.00022 vs 0.0048 ± 0.0005) compared to the LC group. GB treatment also reduced significantly (*p* < 0.05) the DRG level of NFκB (0.0027 ± 0.00056 vs 0.0048 ± 0.0005) compared to LC group (Fig. 3C). The concentration of PGE_2_ in the DRG of LC group was significantly (*p* < 0.05) increased (9.30 ± 0.58) compared to normal (6.15 ± 0.31) and sham (6.17 ± 0.58) control groups (Fig. 3D). However, GS treatment significantly (*p* < 0.05) reduced DRG level of PGE_2_ (6.62 ± 0.52 vs 9.30 ± 0.58) compared to the LC group. CS treatment significantly (*p* < 0.05) reduced DRG level of PGE_2_ (6.59 ± 0.53 vs 9.30 ± 0.58) compared to the LC group. Animals treated with the combination of GS and CS had significantly (*p* < 0.05) reduced level of PGE_2_ in the DRG (6.34 ± 0.67 vs 9.30 ± 0.58) compared to the LC group. Animals treated with GB showed a significant (*p* < 0.05) reduction in the DRG level of PGE_2_ (6.58 ± 0.85 vs 9.30 ± 0.58) compared to LC group (Fig. 3D). Investigation made on the concentration of IL-1β in the DRG of LC animals showed significant (*p* < 0.05) increase (31.35 ± 5.25) compared to sham (15.92 ± 2.74) and non-ligated control (15.67 ± 2.38) animals (Fig. 3E). However, animals treated with GS had significantly (*p* < 0.05) reduced level of IL-1β in the DRG (18.08 ± 0.91 vs 31.35 ± 5.25) compared to the LC group. Animals treated with CS significantly (*p* < 0.05) reduced DRG level of IL-1β (17.56 ± 1.71 vs 31.35 ± 5.25) compared to the LC group. Animals treated with the combination of GS & CS had significantly (*p* < 0.05) reduced level of IL-1β in the DRG (14.85 ± 1.55 vs 31.35 ± 5.25) compared to the LC group. GB treatment showed a significant (*p* < 0.05) reduction in DRG level of IL-1β (14.64 ± 1.35 vs 31.35 ± 5.25) compared to LC animals (Fig. 3E). Investigation of IL-6 concentration in the DRG of LC group showed a significant (*p* < 0.05) reduction (1139.00 ± 34.01) compared to normal (776.02 ± 112.50) and sham (780.40 ± 96.89) animals (Fig. 3F). GS treatment significantly (*p* < 0.05) reduced DRG level of IL-6 (991.70 ± 48.87 vs 780.40 ± 96.89) compared to the LC group. Animals treated with CS showed a significant (*p* < 0.05) reduction in IL-6 level in the DRG (975.60 ± 71.41 vs 780.40 ± 96.89) compared to LC group (Fi. 3F). Animals treated with the combination of GS and CS showed a significant (*p* < 0.05) reduction in the concentration of IL-6 in the DRG (771.40 ± 106.10 vs 776.20 ± 112.5) compared to LC group (Fig. 3F). However, GB treated animals did not produce a significant reduction in the concentration of IL-6 in the DRG compared to LC group (Fig. 3F).

## Immunohistological examination

**Figure 4:**
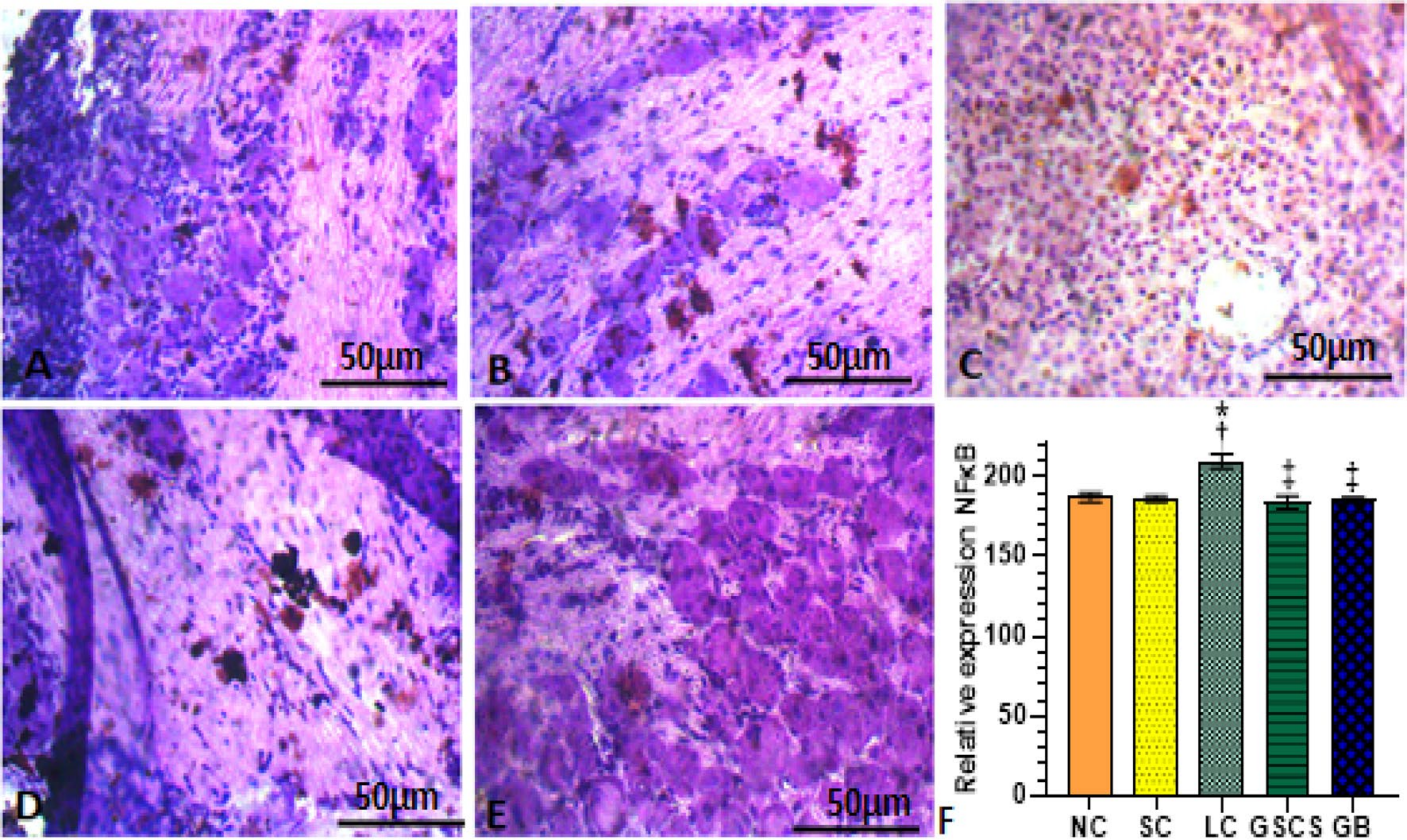
The combination of GS & CS treatment reduced relative expression of NFκB in sciatic nerve CCI model of neuropathic pain. (**A**) NC (**B**) SC (**C**) LC (**D**) GSCS (**E**) GB (**F**) relative expression of NFκB. Values are shown as the mean ± SEM (n = 6 for each group). *†‡*p* < 0.05 *Significant versus NC, **†**Significant versus SC, **‡**Significant versus LC).

**Figure 5:**
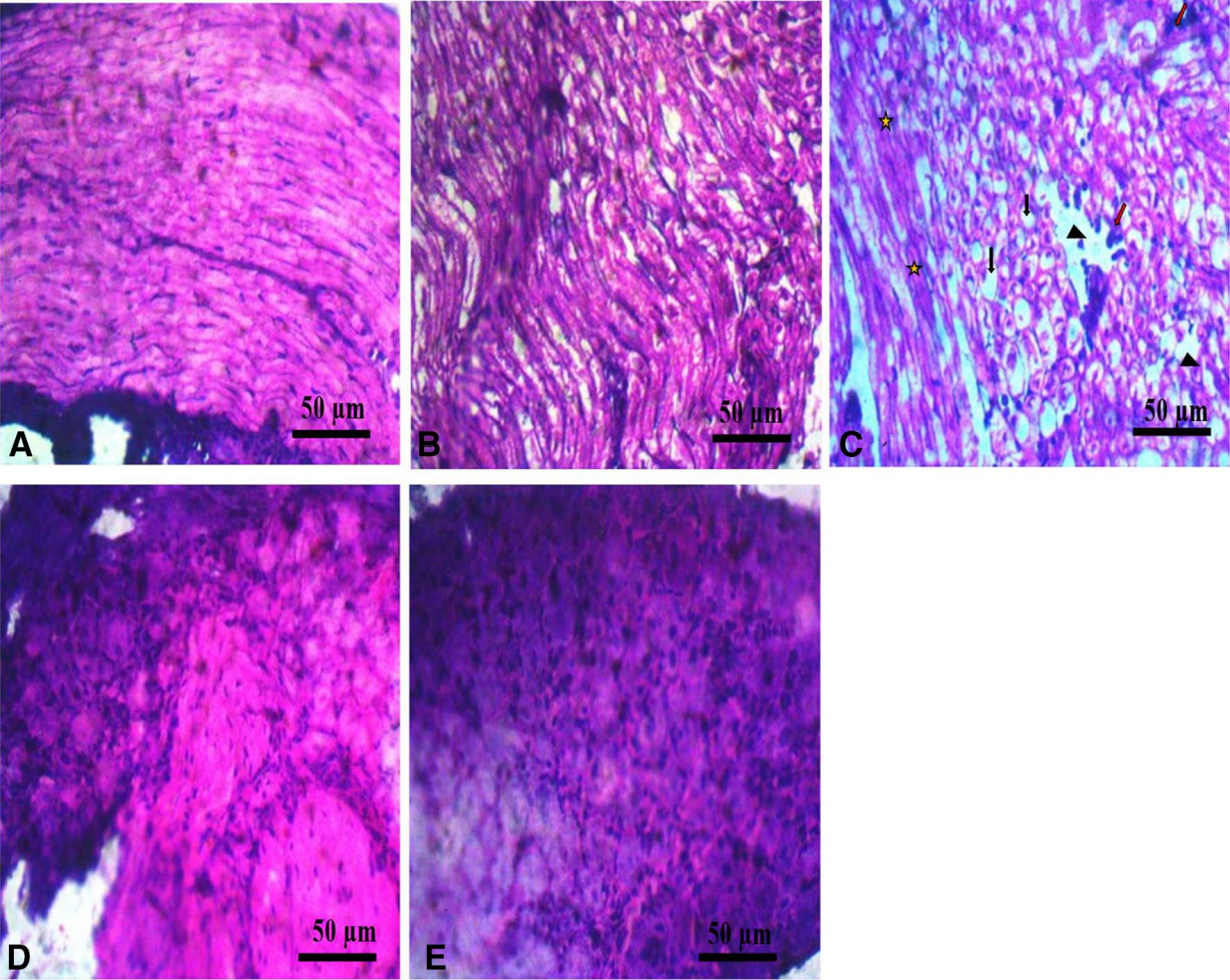
The combination of GS and CS treatment improved neuronal myelination in DRG in CCI model of neuropathic pain. Hematoxylin and eosin (H & E) preparation of lumbar DRG at × 400 magnification. (**A**) NC (**B**) SC (**C**) LC shows diffuse gliosis, edema (arrow head), vacuolation (black arrow), degenerated myelin sheath (star), perineural inflammation (red arrow) (**D**) Co-administration of GS & CS attenuated edema, gliosis, vacuolation, perineural inflammation and myelin sheet degeneration, shows proliferation of glial cells (arrow head) and intact perineural sheet (red arrow) (**E**) GB shows proliferation of glial cells (arrow head) and intact perineural sheet (red arrow).

CCI increased significantly (*p* < 0.05) the expression of NFκB (Fig. 4C) in the DRG of LC group (209.50 ± 4.50) compared to non-ligated (187.00 ± 3.00) and sham (186.00 ± 2.00) control groups. The structural integrity of the DRG axons were compromised and disorganised, with increase vacuolisation, diffuse gliosis, perineural inflammation and edema in the LC group (Fig. 5C). GS and CS treatment significantly (p < 0.05) reduced the expression of NFκB (Fig. 4) in the DRG (184.00 ± 4.00 vs 209.50 ± 4.50) compared to LC group. Also, increase myelination, reduced vacuolisation, reduced edema were observed in the GS & CS treated group (Fig. 5).

## Discussion

Neuropathic pain has become quite a universal burden and concern^11,12^. Sciatic nerve CCI is a well validated neuropathic pain (NP) model and it is often used in screening of possible beneficial agents against NP in animals^13^. This NP model produces pain linked behaviour that is identical to that of humans^14^. As far as we know, no related work has been done on the synergistic effect of GS and CS on CCI and that makes this study the first to investigate the beneficial effect of the combination of GS and CS in CCI model of NP. Neurobahavioural studies like mechanical allodynia (von Frey Filament), cold allodynia (acetone), thermal hyperalgesia (hot plate), sciatic nerve functional index (SFI) were examined respectively. Thermal hyperalgesia and Mechanical allodynia are basic symptoms of neuropathic pain that can be modelled in animal studies^13^. Biochemical parameters in the DRG were also investigated.

This study clearly showed neurohavioural derangement following CCI which resulted into mechanical allodynia, thermal hyperalgesia, cold allodynia and derangement in the sciatic nerve functionality. These derangements were observed on the 3rd day post-CCI and lasted throughout the period of experiment. It has been shown that rats subjected to CCI elicited mechanical allodynia, cold allodynia and thermal hyperalgesia^15,16^. However, treatments with GS and CS resulted in a reduced response to pain behaviour by suppressing mechanical allodynia, cold allodynia and thermal hyperalgesia by increasing pain thresholds. Interestingly, the combination of GS and CS treatment resulted in a synergistic reduction in pain behavioural response. In this study we observed that the combination of GS and CS showed similar efficacy as GB in suppressing pain behaviours. CCI led to a prominent loss of the hind limb function of the ipsilateral paw. This was evaluated via SFI based on the analysis of foot print. Abnormal gait was observed in CCI animals. This is said to be due to degradation of myelin sheath resulting to a decrease in the density of myelinated nerves which in turn negatively affects bearing as shown^17,18^. Myelinated neuronal loss and anomaly in posture seen after CCI induction may be far-fetched from neuropathic pain linked oxidative stress and neuroinflammation^18^.

It has been emphasised numerous times that cytokines are signalling proteins that are crucially involved in the nervous system response to nerve injury. They function as intercellular mediator in response to nervous system injury^19,20^. Recently conducted experimental studies over the years have proven beyond doubt that proinflammatory cytokines can bring about or aid NP^21^. Differently, in animal model, administering anti-inflammatory drugs or cytokines or blockade of proinflammatory cytokines have lessen NP^22,23^. The evaluation of DRG neuropathic pain markers revealed that treatments with GS, CS, the combination of GS and CS and treatment with GB reversed the concentration of NGF, PGE_2_, TNF-α, IL-1β, Glutamate and CGRP. IL-6 was only reversed with the combination of GS and CS treatment. In an immunomodulatory and anti-inflammatory study in osteoarthritis, it was reported that CS a member of the GAGs exerts its beneficial effect by suppressing the generation of pro-inflammatory mediators^24,25^. It was also reported in a non-neuropathy study on cell culture experimentation that glucosamine sulphate reduced the generation of pro-inflammatory mediators by suppressing the production of ROS and NFkB activation^26,27^.

A previous report showed that NGF (a member of the neurotrophins (NTs) family) plays a crucial role in the pathobiology of neuropathic pain^28^. NGF are synthesised mainly in the DRG and modulate central sensitisation by binding with neurotrophic tyrosine kinase A (TrkA) receptor and pan neurotrophin receptor at 75 kDa (p75NTR)^29^. The interaction of NGF with TrkA receptor results to signalling that activates the upregulation of transient receptor potential vanilloid 1 (TRPV1), CGRP, substance-P (SP), sodium channels (Na_v_1.8 and Na_v_1.9) expression^28^. The interaction of NGF with p75NTR results to downstream signalling linked with NP via the c-Jun and NF-kB pathway. Also, activation of p75NTR can trigger Trk mediated signalling in NP^28^. This study showed that GS and CS and the combination of GS and CS treatments suppressed NGF level in the DRG and showed similar efficacy as GB in reducing NGF level in the DRG.

TNF-α has been reported to be involved in early degenerative changes in peripheral nerve injury^30^. These molecules are formed by Schwann cells in Wallerian degeneration process^31^ and are reported to contribute to hyperalgesia and allodynia^32–34^. Furthermore, TNF-α acutely elevates Tetrodoxin-resistant (TTX-R) Na^+^ currents^35^. This modulation occurs through the activation of tumor necrosis factor receptor 1 (TNFR1) and phosphorylation of p38 (MAPK)^35^. Hence, this study showed that suppression of TNF-α following treatment with GS, CS and the combination of GS and CS reveals the beneficial effect of these GAGs in reducing mechanical allodynia, cold allodynia and thermal hyperalgesia. TNF-α activity has been postulated to cause an increase in primary afferent neuron excitability via reduction in currents in potassium channels or increase in currents through sodium or calcium channels^35,36^. NFκB, a family of DNA-binding proteins that are related in structure are variously involved in many pathological and physiological processes which include neurodegenerative diseases^37^ and chronic pain^38^. Activation of NFκB involves two different pathways, which are the classical or canonical and the alternative or non-canonical pathways^39^. Evidences have shown that TNF-α is crucial in both pathways^39^. Past studies have reported that TNF-α/NFκB signalling activation results to persistent hyperexcitability of neurons in the DRG^40^ via upregulation of Na^+^ channels^41^. From this study, the level of NFκB in the DRG of CCI rats were elevated. Treatments with GS, CS and the combination of GS and CS suppressed NFκB level in the DRG. It is therefore suggested that the suppression of TNF-α in this study might be responsible for the reduced expression of NFκB observed. Cyclooxygenase (COX) enzymes (COX 1 and COX 2) have been implicated to play a crucial role in arachidonic acid pathway involved in pain exacerbation. The prostaglandins derived from arachidonic acid heightened neuronal excitability, generate and transmit pain signals. It has been confirmed that COX play an intricate role in the downstream signalling of PGE_2_^42^. The same study confirmed that treatments with COX inhibitors suppresses the release of PGE2 in the DRG^42^. Studies using localised stimulation showed that injected PGE_2_ reduced von Frey filament threshold for mechanical allodynia^43^. It was also reported that peripheral nerve injury resulted to increase in PGE_2_ level in the lumbar DRG and that proinflammatory cytokine IL1-β aided production of PGE_2_ in DRG neurons. Under physiologic circumstances, IL1-β production is at low levels in the spinal cord. Following injury to the peripheral nerve, the level of IL1-β expression is upregulated^44^. This presented study showed that the level of IL1-β in the DRG of CCI rats was upregulated and treatments with GS and CS reduced expression of IL1-β. It is interesting to note that suppression of IL1-β might be responsible for the reduction in the level of PGE_2_ observed. Our findings showed that IL1-β expression can result to the activation of proinflammatory molecules such as IL-6, SP, PGE_2_ through complex signalling cascades^44–46^. Evidence has shown that IL1-β just like NGF can modulate excitability of neurons via TRPV1, GABA receptors, sodium channels and NMDA receptors^34^.

Data from this study revealed that the level of IL-6 in the DRG of CCI rats was elevated. Studies have shown that IL-6 is crucial in the nervous system response to injury^47,48^. IL-6 is implicated in the survival of neurons, protection of the neuron against damage^47,48^ and pain modulation^49,50^. The level of IL-6 in the DRG is upregulated following CCI^51,52^. It was reported that the intrathecal injection of IL-6 into the hind paw of rats brought about touch-evoked hyperalgesia^53^ and mechanical allodynia^54^. These research findings suggest that IL-6 may centrally play a key role in the event cascades that leads to NP. The level of IL-6 was however reduced following treatment with the combination of GS and CS. Immunohistochemistry and histopathological study showed that the combination of GS and CS treatment reduced oedema, vacuolation, perineural inflammation and gliosis, increased proliferation of glial cells and improved overall integrity of the DRG. This improvement exerted by GS and CS was similar to that exerted by GB. GS and CS which are two separate compounds exhibited analgesic effects when administered individually. Also, the combination of both GS and CS interact additively thereby bringing about an analgesic effect which was significantly greater than the analgesic effect of the individual drugs. This significant analgesic effect of the combination therapy over the individual drugs is thought to be synergistic.

## Conclusions

This is the first study to investigate the therapeutic role of CS, GS and the combination of both GS and CS on DRG proinflammatory cytokines in a CCI model of neuropathic pain. This study has shown that CS, GS and the combination of both GS and CS can reduce mechanical allodynia, cold allodynia and thermal hyperalgesia, improve DRG neuronal functions and as well improve sciatic nerve functional index. The efficacy of CS and GS in this model is through suppression of IL1-β, NFκB, NGF, TNF-α, IL-6, PGE_2_. This study therefore suggests that the combination of GS and CS may have synergistic therapeutic potential in managing injury to the peripheral nerve.

## Materials and methods

### Animals

All experimental procedures were approved by the University of Ilorin Ethical Review Committee (Approval Number UERC/ASN/2019/1949) and conducted in accordance with ARRIVE guidelines. Male Wistar rats weighing 150–200 g, were used for the study and were housed in the Faculty of Basic Medical Sciences, University of Ilorin animal house. The rats were acclimatised for 2 weeks after which the experiments commenced. The animals were housed in cages made of wood, exposed to 12 h light/dark cycle and had unhindered access to water and food with exception to experimental procedure periods. All experiments were performed between 8am and 2 pm to avoid possible diurnal variation in the behavioural tests. Pain tests or neurobehavioural test were carried out by experimenters blinded to the treatments. All efforts to minimise animal suffering and to use the minimum number of animals required to produce dependable results were employed. The animals were handled based on the laid down principle of the Guide for the Care and Use of Laboratory Animals published by National Institute of Health.

### Drugs and chemicals

Analytical grade glucosamine sulphate and chondroitin sulphate (Jiaxing Hengjie Biopharmaceutical Co., Ltd, China) were used. These drugs and their combinations were dissolved in 0.9% NaCl and orally administered using oral cannula. sodium pentobarbital (50 mg/kg i.p.) (Sigma-Aldrich, USA) was used as anesthetic agents. Other chemicals and materials were obtained from Bridge Biotech Ltd, Nigeria. 4.0 silk suture was used to induce CCI and suture the muscles, while 4.0 nylon suture was used to suture the skin.

### Experimental design

Animals were randomly grouped into seven (7) groups of six rats (n = 6) per group with sample size determined based on G*power analysis^55^ and the ethical concept of reduction^56^. The grouping (Fig. 1A) consists of four (4) control groups.

The non-ligated control (NC) animals received normal saline (2.5 ml/kg oral) treatment without ligation. The sham control (SC) animals had the skin and muscle opened and sutured without nerve ligation. The sham animals were treated with 2.5 ml/kg orally administered normal saline. The CCI rats or the ligated control (LC) animals were induced with right sciatic nerve CCI and were treated with 2.5 ml/kg orally administered normal. GS treated animals underwent surgical procedure with chronic constriction of the right sciatic nerve and were treated with GS (240 mg/kg oral). CS treated animals underwent surgical procedure with chronic constriction of the right sciatic nerve and were administered oral CS (900 mg/kg oral*.*). GSCS treated animals underwent surgical procedure with CCI of the right sciatic nerve following which they were administered oral combination of GS (240 mg/kg) and CS (900 mg/kg). GB treated rats underwent surgical procedure with CCI of the right sciatic nerve and were treated with 60 mg/kg orally administered gabapentin (a reference control).

**Figure 6:**
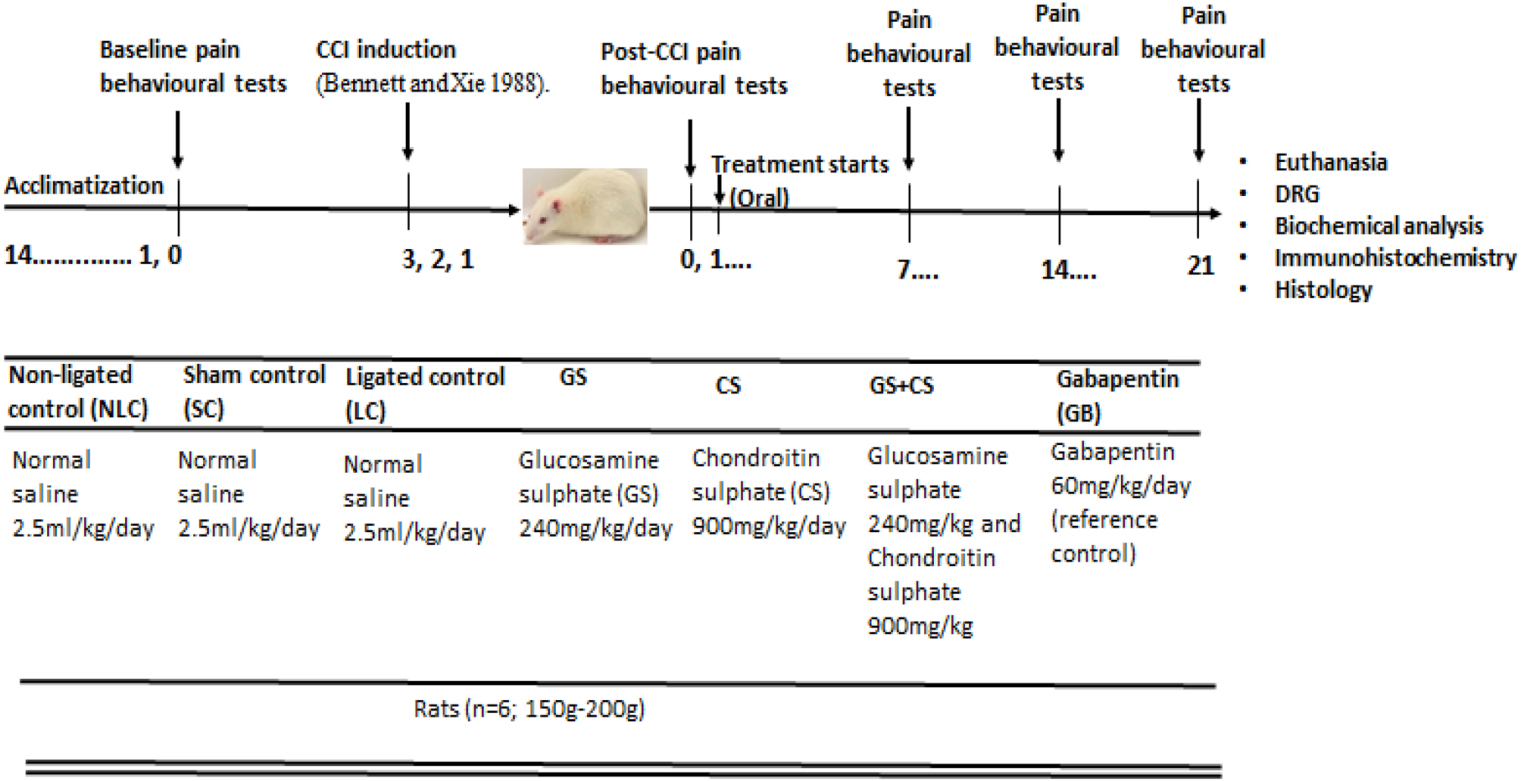
Schematic representation of animal grouping, treatments, CCI induction, drug treatments period and neurobehavioural tests schedule.

The treatments duration lasted for 21 days which commenced on the 3rd day post CCI intervention (Fig. 6).

### Induction of chronic constriction injury

CCI model for neuropathic pain was applied to bring about neuropathy as portrayed by Bennett and Xie^57^. Under sodium pentobarbital anesthesia, incision was made on the skin of the right hind limb and the sciatic nerves underneath the biceps femoris were exposed and freed of adhering tissue and three loose ligatures of silk 4.0 were tied around it with 3 mm gap approximately. The sham control rats had the skin and femoris muscle opened and sutured without nerve ligation. To examine the synergistic effects, we administered a combination of GS (240 mg/kg) and CS (900 mg/kg) and investigated the effects on thermal hyperalgesia, mechanical allodynia, cold allodynia, sciatic nerve functional index and central proinflammatory markers.

### Mechanical allodynia (von Frey Test)

To investigate the sensory functionality of the sciatic nerve, von Frey filament test (Ugo Basile, Italy) was used to measure paw withdrawal threshold to mechanical stimuli. The rats were housed in a transparent plastic with a mesh floor made of metal and before testing, were habituated to the environment for about 10 min. von Frey filaments of evaluator sizes 4.17, 4.31, 4.56, 4.74, 4.93, 5.07, 5.18, 5.46, 5.88 and 6.1 (corresponding to forces of 1.4 g, 2 g, 4 g, 6 g, 8 g, 10, 15 g, 26 g, 60 g, 100 g respectively) were applied to the hind paw’s plantar surface. Each filament was used five times for a single paw and the mechanical threshold was calculated based on the method of Bonin et al.^58^.

Mechanical allodynia was determined prior to CCI (baseline), then 3, 10, 17 and 24 days following CCI. Drugs treatment commenced on day 3 following CCI after post CCI tests.

### Thermal hyperalgesia (Hot-Plate Test)

Thermal sensitivity of the hind paw was evaluated with a hot-plate apparatus (Hefe-joyce, China). The hot plate was preheated to 55 ± 0.5 °C and animals were placed on the preheated plate surface. The reaction time of paw withdrawal was recorded with a cut-of time set at 20 s. Thermal analgesia was determined prior to CCI (baseline), then 3, 10, 17 and 24 days following CCI.

### Cold allodynia (Acetone Test)

Latency of paw withdrawal to cold stimulus was investigated using acetone spray test as described by Yoon et al.^59^. Cold allodynia was determined prior to CCI (baseline), then 3, 10, 17 and 24 days following CCI.

### Histological analysis

Five-ten hours after the last dose of drug treatment, rats were made unconscious and the sciatic nerve proximal to the bifurcation point was dissected. All animals were anaesthetized with sodium pentobarbital. Formalin (10%) was used to fix the DRG, included in paraffin and put through to 7 μm thick transverse sections, followed by further hematoxylin and eosin (H&E) staining. The slides were examined by a person blinded to the treatments. The following parameters were analyzed from the slides: nerve constituents, which include endoneurium, perineurium and epineurium, the nerve fiber, inflammatory cell infiltrates and Schwann cells.

### Biochemical analysis of the DRG

Five to ten hours after the last dose of treatment, the spinal column was dissected from the base of the skull to the level of the femurs, it was cut down the mid-line and the spinal cord extruded and meninges removed as described by Sleigh et al*.*^60^, L3&L4 DRG were extracted and rinsed and stored in ice-cold phosphate buffer solution (PBS) and frozen for biochemical analysis. DRG was homogenized with the aid of a glass homogenizer at 4 °C in ice-cold saline (2 ml) (11 mmol L^−1^ Tris buffer, pH 7.4). Homogenates were centrifuged at 5,000 RPM for ten minutes and supernatants were collected for the estimation of biochemical parameters using ELISA principle.

### Evaluation of proinflammatory mediators

Prostaglandin E_2_ (PGE_2_) (Bioassay Technology Laboratory, Shanghai, China), Tumor necrotic factor alpha (TNF-α) (Elabscience Biotechnology Inc., Texas, USA), interleukin 6 (IL-6) (Elabscience Biotechnology Inc., Texas, USA), interleukin 1 beta (IL1-β) (Elabscience Biotechnology Inc., Texas, USA), nerve growth factor (NGF) (Bioassay Technology Laboratory, Shanghai, China) and nuclear factor kappa B-105 (NFκB105) (Elabscience Biotechnology Inc., Texas, USA) levels in the DRG homogenate were assessed using ELIZA techniques based on the manufacturer’s instructional manual.

### Histological evaluation

DRG tissue (L5) was investigated using haematoxylin and eosin stain (H&E). Histological examination of slide was as described by Windsor^61^ and Hopwood^62^. The DRG tissues were fixed in 10% formalin for over 24 h before dehydrating, clearing, embedding and staining.

### Immunohistochemistry

Expression of nuclear factor kappa B (NFκB p65) was evaluated using the Thermo Scientific Pierce Peroxidase IHC Detection Kit (36,000, Thermo Scientific, USA) with modest procedure modification. Endogenous peroxidase activity was quenched by incubating tissue for 30 min in Peroxidase Suppressor, washed three times in Wash Buffer. Blocking buffer was added to the slides and incubated for 30 min. Excess buffer was blotted from the tissue sections, before addition of primary antibodies; NFkB p65 (Cat #14-6731-81, Thermo Scientific, USA), at a dilution of 1:100 and left overnight in a humidified chamber at 4 °C. Afterward, slides were washed two times for 3 min with Wash Buffer.

The tissue sections were treated with Biotinylated Secondary Antibody and incubated for 30 min. The slides washed three times for 3 min each with Wash Buffer, treated and incubated with the Avidin/Streptavidin-HRP for another 30 min, and washed three times for 3 min each with Wash Buffer. The tissues were incubated with Metal Enhanced DAB (3.3 diaminobenzidine) Substrate Working Solution for 5 min for desired staining to be achieved. The slides were rinsed with distilled water and drained. Adequate amount of Mayer’s hematoxylin stain was dropped on to the slide to cover the entire tissue surface and incubated for 1–2 min at room temperature. Drained off the hematoxylin and washed slide several times with distilled water. The slides were mounted and photomicrographed with Amscope MU900 digital camera attached to the microscope. The intensity of staining was examined with the open-source Fiji (ImageJ) software.

### Statistical analysis

GraphPad Prism 8.0.1 software (USA) was used for statistical analyses. Values are expressed as Mean ± S.E.M. Behavioural data were analyzed using one way and two-way analysis of variance (ANOVA) with Tukey’s post hoc multiple comparisons test. Biomarkers data were analyzed with one-way ANOVA followed by Turkey’s post hoc multiple comparisons test *p* < 0.05 are considered significant.

## Data Availability

The data supporting the findings from this study are available on request from the corresponding author.
